# 

**Published:** 1942

**Authors:** 


### The Bristol
### Medico-Chirurgical Journal

A JOURNAL OF THE MEDICAL SCIENCES FOR THE
WEST OF ENGLAND AND SOUTH WALES

PUBLISHED UNDER THE AUSPICES OF
*THE BRISTOL MEDICO-CHIRURGICAL SOCIETY*

EDITOR
J. A. NIXON, C.M.G., M.D., F.R.C.P.

WITH WHOM ARE ASSOCIATED
E. W. HEY GROVES, D.Sc., M.S., F.R.C.S.
A. E. ILES, O.B.E., M.B., B.S., F.R.C.S.
A. RENDLE SHORT, M.D., B.S., B.Sc., F.R.C.S.
W. MELVILLE CAPPER, M.B., B.S., F.R.C.S.
E. WATSON-WILLIAMS, M.C., Ch.M., F.R.C.S., Assistant Editor

"Scire est nescire, nisi id me

Scire alius sciret"

*VOL. LIX*

BRISTOL: J. W. ARROWSMITH LTD.

### Contents of Vol. LIX.

*Page.*

Inunction of Testosterone in Mental Disease. By A. Guirdham, D.M., B.Sc., D.P.M. .. .. .. .. .. .. .. .. 1

The Fundamental Principles of Fracture Treatment. By K. H. Pridie, F.R.C.S. .. .... .. .. .. .. .. .. .. 7

Experiences of Typhus Fever. By F. Kohn, M.D. .. .. .. 13

The Bristol Scheme for Dealing with an Outbreak of Typhus. By R. J. Irving Bell, M.R.C.S., L.R.C.P., M.D. .. .. .. .. 16

The Thirty-first Long Fox Memorial Lecture: The Experimental Study of Development. By Professor C. M. Yonge, D.Sc. .. 49

Hospital Re-organization. By Somerville Hastings, M.S., F.R.C.S. 61

Anæsthesia .. .. .. .. .. .. .. .. .. 19

The Story of the Bristol Hospitals .. .. .. .. .. 66

Reviews of Books .. .. .. .. .. .. .. 23, 71

Editorial Notes .. .. .. .. .. .. .. 27, 73

Obituaries .. .. .. .. .. .. .. .. .. 77

The Medical Library of the University of Bristol .. 33, 79

Publications Received .. .. .. .. .. .. 38, 82

Local Medical Notes .. .. .. .. .. .. 39, 82

### The Medical Library of the University of Bristol

WITH WHICH IS INCORPORATED

**The Library of the Bristol Medico-Chirurgical Society.**

---

*The following donations have been received since the publication of the list in November,* 1941.

June, 1942.

| | |
|---|---|
| Brompton Hospital (1) .. .. .. .. .. | 1 volume. |
| Commonwealth Fund (2).. .. .. .. .. | 1 volume. |
| Professor E. Fawcett (3).. .. .. .. .. | 5 volumes. |
| General Medical Council (4) .. .. .. .. | 1 volume. |
| Professor E. W. Hey Groves (5) .. .. .. | 2 volumes. |
| Dr. C. B. Lavalle (6) .. .. .. .. .. | 1 volume. |
| Bristol Medico-Chirurgical Society (7) .. .. .. | 3 volumes. |
| Michell Clarke Fund (8) .. .. .. .. .. | 11 volumes. |
| Munro Smith Fund (9) .. .. .. .. .. | 2 volumes. |
| Dr. Nierenstein (10) .. .. .. .. .. | 1 volume. |
| Dr. J. A. Nixon (11) .. .. .. .. .. | 1 volume. |
| University of Otago (12).. .. .. .. .. | 1 volume. |
| St. Bartholomew's Hospital (13) .. .. .. | 1 volume. |
| Mrs. Sarsfield (14).. .. .. .. .. .. | 4 volumes. |
| Shaw Bequest (15) .. .. .. .. .. | 2 volumes. |
| Dr. Stengel (16) .. .. .. .. .. .. | 30 volumes. |
| Messrs. John Wright & Sons Ltd. (18) .. .. | 1 volume. |

Unbound periodicals have also been received from Dr. S. B. Adams, Dr. R. F. Barbour, Professor E. W. Hey Groves, Dr. C. B. Lavalle, Dr. T. Milling, Professor C. Bruce Perry, Dr. Stengel, Dr. J. Odery Symes, Dr. C. H. Walker, Messrs. John Wright & Sons Ltd.

### THE ONE HUNDRED AND SEVENTY-FIFTH LIST OF BOOKS.

The figures in round brackets refer to the figures after the names of the donors. The books to which no such figures have been attached have either been bought from the Library Fund or received through the *Journal*.

| | | |
|---|---|---|
| **Bamford, F.** | *Poisons, their isolation and identification* | 1940 |
| **Barnard, W. G.** | *Elementary Pathological Histology* 2nd Ed. | 1940 |
| **Bergler, E.** | *Die psychische Impotenz des Mannes* (16) | 1937 |
| **Bigger, J. W.** | *Handbook of Bacteriology* 5th Ed. | 1939 |
| **Bourne, A. W.** | *Synopsis of Obstetrics and Gynæcology* 8th Ed. (8) | 1941 |
| **Browne, R. C., and Gilbert, B.** | *Midwifery* (8) | 1940 |
| **Buchan, W.** | *Domestic Medicine* 19th Ed. (11) | 1805 |
| **Bühler, C.** | *Der Menschliche Lebenslauf als psychologisches Problem* (16) | 1933 |
| **Bunting, R. W., and Hill, I. J.** | *Textbook of Oral Pathology.* 2nd Ed. | 1940 |
| **Clark, A. J.** | *Applied Pharmacology* 7th Ed. | 1940 |
| **Commonwealth Fund** | *Annual Report,* 1941 | 1942 |
| **Craig, M., and Beatin, I.** | *Psychological Medicine* 4th Ed. | 1926 |
| **Cross, H. H. U.** | *Electricity in Therapeutics: A Technical and Clinical Compendium* (14) | 1936 |
| **Cushny, A. R.** | *Pharmacology and Therapeutics* 12 Ed. | 1941 |
| **Fahrenkamp, K.** | *Wesentliches und Altägliches vom Herzkranken* (16) | 1936 |
| **Feer, E.** | *Lehrbuch der Kinderheilkunde* (16) | 1920 |
| **Goodman, L., and Gilman, A.** | *Pharmacological Basis of Therapeutics* | 1941 |
| **Gray, H.** | *Anatomy, Descriptive and Surgical* (17) | 1858 |
| **Green, T. H.** | *Manual of Pathology* 16th Ed. (8) | 1940 |
| **Harris, D. T.** | *Technique of Ultra-Violet Radiology* (14) | 1932 |
| **Hentschel, C. C., and Cook, W. R. I.** | *Biology for Medical Students.* 3rd Ed. | 1941 |
| **Hill, D., and Howitt, F.** | *Insulin* (10) | 1936 |
| **Höber, R.** | *Lehrbuch der Physiologie des Menschen* (16) | 1920 |
| **Holt, L. E., and Howland, J.** | *The Diseases of Infancy and Childhood* 11th Ed. (8) | 1940 |
| **Hutchison, R.** | *Food and Principles of Dietetics.* 9th Ed. (9) | 1941 |
| International Medical Congress. | *Album of* 16*th Congress* (3) | 1909 |
| **Jackson, D. E.** | *Experimental Pharmacology and Materia Medica* 2nd Ed. | 1939 |
| **Jackson, H.** | *Memorial Volume,* 1935 (16) | 1935 |
| **Joslin, E. P.** | *Treatment of Diabetes Mellitus* 7th Ed. (8) | 1940 |
| **Kanders, O.** | *Zur Klinik und Analyse der Psychoamotorischen Störung* (16) | 1931 |
| **Kovács, E. R.** | *Electrotherapy and the Elements of Light Therapy* (14) | 1932 |
| **Kraus, F., and Brugsch, I., hrsg.** | *Spezielle Pathologie und Therapie innerer Krankheiten.* II, 1—3; X, 2—3 (16) | 1919–24 |
| **Kronfeld, A.** | *Das Wesen Psychiatrischen Erkenntnis* (16) | 1920 |
| **Lavalle, C. B.** | *Biological Surgery in Bone-Tuberculous Patients* (6) | 1941 |
| **Lavalle, C. B.** | *Tuberculosis Quirurgica* (6) | 1938 |
| **Lewin, P.** | *The Foot and Ankle* 2nd Ed. (5) | 1941 |

| | | |
|---|---|---|
| **Linell, E. A.** | *Distribution of Nerves in the Upper Limb* (Thesis) .. .. .. .. .. .. .. .. (3) | 1940 |
| **Lyle, K., and Jackson, S.** | *Practical Orthoptics in the Treatment of Squint* 2nd Ed. (7) | 1940 |
| **McCoy, J. D.** | *Applied Orthodontics* .. .. .. .. .. 5th Ed. | 1941 |
| **McDougall, W.** | *Psychopathologie* .. .. .. .. .. .. (16) | 1931 |
| **MacKee, G. M.** | *X-rays and Radium in the Treatment of Diseases of the Skin* .. .. .. 3rd Ed. (8) | 1938 |
| **Martindale, W.** | *Extra Pharmacopœia.* Volume I 22nd Ed. | 1941 |
| **Meakins, J. C.** | *Practice of Medicine* .. .. 3rd Ed. (8) | 1941 |
| **Muir, R.** | *Textbook of Pathology* .. .. .. .. 5th Ed. | 1941 |
| **Naito, I.** | *Das Hirnrindenbild bei Schizophrenie* (16) | 1924 |
| **Newell, R. L.** | *The Anatomy and Histology of the Prostate* (3) | 1921 |
| **Orr. H. W.** | *Wounds and Fractures* .. .. .. (5) | 1941 |
| **Panton, J. A.** | *Factors bearing upon the Etiology of Femoral Hernia* .. .. .. .. .. .. .. .. (3) | 1921 |
| **Potzl, O.** | *Die Aphasielehre vom Standpunkte der Klinischen Psychiatrie* .. .. .. (16) | 1928 |
| **Power, D., and Waring, H. J.** | *A Short History of St. Bartholomew's Hospital, 1123–1923* .. .. .. .. .. .. .. (13) | 1923 |
| **Price, F. W.** (Ed.) | *Textbook of the Practice of Medicine.* 6th Ed. (8) | 1941 |
| **Reinhold, J.** | *Wissenschaftliche Festschrift zum 50. Geburtstage* .. .. .. .. .. .. .. (16) | 1936 |
| **Rogers, L., and Megaw, J. W. D.** | *Tropical Medicine* .. .. 4th Ed. | 1942 |
| **Rolleston, H.** | *British Encyclopædia of Medical Practice. Surveys and Abstracts,* 1939 .. .. .. .. | 1940 |
| **Rorschach, H.** | *Psychodiagnostik* .. .. .. .. .. .. (16) | 1932 |
| **Ross, I. S., and Fairlie, H. P.** | *Handbook of Anæsthetics* 5th Ed. (8) | 1941 |
| **Ross, T. A.** | *Lectures on War Neuroses* .. .. .. (8) | 1941 |
| **Roxburgh, A. C.** | *Common Skin Diseases* .. .. 6th Ed. (7) | 1931 |
| **Rowzee, L.** | *The Queen's Wells* .. .. .. .. .. .. .. | 1671 |
| **Sarason (hrsg.)** | *Der Schlaf* .. .. .. .. .. .. .. .. (16) | 1929 |
| **Schliephake, E.** | *Short-Wave Therapy.* English translation by R. K. Brown .. .. 2nd English Ed. (14) | 1928 |
| **Schwarz, O.** | *Medizinische Anthropologie* .. .. (16) | 1929 |
| **Schwarz, O.** | *Psychogenese und Psychotherapie. Körperlicher Symptome* .. .. .. .. .. .. .. (16) | 1925 |
| **Science Museum** | *Handlist of Short Titles of Current Perodicals in the Science Library.* Pt. I, Alphabetical 5th Ed. First Supplement. 5th Ed. | 1938–9 |
| **Shaw, W.** | *Textbook of Gynæcology* .. 3rd Ed. (8) | 1941 |
| **Sheldon, W.** | *Diseases of Infancy and Childhood* 3rd Ed. (8) | 1941 |
| **Sigerist, H. E.** | *Medicine and Human Welfare* .. .. .. .. | 1941 |
| *A Sketch of the Origin of "The Retreat," an Institution near York* .. .. | | 1828 |
| **Stewart, C. P., and Dunlop, D. M.** | *Clinical Chemistry in Practical Medicine* 2nd Ed. | 1937 |
| **Strumpel, A.** | *Lehrbuch der Speziellen Pathologie und Therapie.* 2 Bde.. .. .. .. .. .. .. (16) | 1922 |
| **Sutherland, D. M.** | *The Anatomy of the Seminal Vesicles with reference to the Treatment of Seminal Vesiculitis* (Typescript) .. .. .. .. .. (3) | 1921 |

| | | | |
|---|---|---|---|
| **Swammerdam, J.** | *Miraculum Naturae sive Uteri Muliebris Fabrica* | | 1679 |
| **Tidy, N. M.** | *Massage and Remedial Exercises* | 5th Ed. (7) | 1941 |
| **Tigerstedt, R.** | *Lehrbuch der Physiologie des Menschen,* Bd. II | (16) | 1920 |
| **Tilney, F.** | *The Brain from Ape to Man.* 2 vols | (15) | 1928 |
| **Triepel, H.** | *Lehrbuch der Entwicklungsgeschichte* | (16) | 1917 |
| **Van der Heide, C.** | *Klinisch Anatomische Studie over Piksche Ziekte* | (16) | 1934 |
| **Vaughan, H. S.** | *Congenital Cleft Lip, Cleft Palate, and Associated Nasal Deformities* | | 1940 |
| **Vernon, H. M.** | *Health and Efficiency of Munition Workers* | | 1940 |
| *Virus and Rickettsial Diseases* | | (9) | 1941 |
| **White, R. P.** | *The Dermatergoses or Occupational Affections of the Skin* | 4th Ed. | 1934 |
| **Whitla, W.** | *Pharmacy, Materia Medica, and Therapeutica* | 13th Ed. | 1939 |
| *Wiener Allgemeines Krankenhaus.* 1784–1934. *Gedenkschrift* | | (16) | 1935 |
| **Weininger, O.** | *Geschlecht und Charakter* | (16) | 1921 |
| *Year Book of Radiology,* 1941 | | (18) | 1941 |
| **Zuisser and Bayne Jones** | *Textbook of Bacteriology* | 8th Ed. | 1939 |

### TRANSACTIONS, REPORTS, JOURNALS, ETC.

| | | |
|---|---|---|
| Brompton Hospital Reports. Vol. X, 1941 | (1) | 1941 |
| Dentists' Register, 1942 | (4) | 1942 |
| Jahrbücher fur Psychiatrie und Neurologie. Vol. 47, Parts 3/4, Vol. 54, Parts 1/2. | | |
| Medical Directory, 1942 | | 1942 |
| Proceedings of the University of Otago Medical School, Number 19 | (12) | 1942 |
| Quarterly and Cumulative Index Medicus. Vol. 29. 1941. 2 vols. | | 1941 |

---

### MISSING BOOKS.

The following books are missing from the Library, borrowed, it is thought, by non-student members. It is hoped that anyone possessing any of these volumes will return them to the Library as soon as possible :—

| | |
|---|---|
| **Allen** | *Diseases of Children.* |
| **Buckmaster and Hickman** | *Practical Physiology.* |
| **Cunningham** | *Manual of Practical Anatomy.* I. |
| **Barcroft** | *Architecture of Physiological Function.* |
| **Gray** | *Textbook of Anatomy.* 27th Ed. |
| **Hall** | *Diseases of the Ear, Nose and Throat.* |
| **Grincker** | *Neurology.* |
| **Mercer** | *Orthopædic Surgery.* |
| **Melland** | *Pocket Book of Clinical Suggestions.* |
| **Ormerod** | *Diseases of the Nervous System.* |
| **Snell** | *Examination of the Eye.* |
| **Vazifdar** | *Physiology of the Nervous System.* |
| **Wyard** | *Handbook of Diseases of the Stomach.* |

### THE "PRACTITIONER."

The Medical Library is in need of Parts 5 and 6 of Volune 147 (November and December, 1941) to complete the set. These are out of print at the publishers. If any reader is able to supply the parts they would be gladly received by The Librarian at the University.

---

### NOTICE.

It may not be generally known that it is possible to borrow from other Libraries both books and periodicals which may not be in the Libraries of the University. These loans are obtained sometimes by direct application to other Universities or Medical Libraries, and sometimes by application to the National Central Library, which itself borrows from many "outlier" Libraries from which direct borrowings are not possible. It is not intended that this scheme of Library co-operation which, in peace time, includes European and American Libraries, should be used to obtain recent inexpensive books or current parts of periodical publications. Any requests for such loans should be sent to The Librarian, or passed to the Assistant-in-charge in the Library, giving full details as to Author, title and date of publication, and in the case of periodicals, the volume number, year of publication, together with the author and title of the article.

### Publications Received

From Messrs. George Allen & Unwin, Ltd. :

*A Practical Method of Self-Analysis.* By Farrow. Price 6s.

From Messrs. Cassell & Co. Ltd. :

*Diseases of the Eye.* By Eugene Wolff. Price 21s.

From Messrs. H. K. Lewis & Co. Ltd. :

*Cardiac Symptoms in the Neuroses.* By D. M. Baker. Price 4s. 6d.

*Materia Medica for Nurses.* By A. Muir Crawford, M.D., F.R.F.P.S.G. Price 4s. 6d. net.

*The Sanitary Inspectors Handbook.* A Manual for Sanitary Inspectors and other executive Public Health Officers. By Henry H. Clay, F.R.San.I., F.I.S.E. Fifth Edition. Price 18s. net.

From Oxford University Press (Oxford War Manuals) :

*The Treatment of Shock.* By R. W. Raven, F.R.C.S., Major R.A.M.C. Price 5s.

*Amputations and Artificial Limbs.* By R. D. Langdale-Kelham, and George Perkins. Price 5s.

*Tropical and Sub-tropical Diseases.* By C. H. Barber, D.S.O., M.A., D.M.(Oxford), M.R.C.S., L.R.C.P. Price 5s.

From Oxford University Press :

*Adolescent Spondylitis or Ankylosing Spondylitis.* By S. Gilbert Scott. Price 15s.

*Principles and Practice of Cardiology.* By J. C. Bramwell and King. Price 35s.

From Messrs. John Wright & Sons Ltd. :

*British Journal of Surgery.* No. 114. October, 1941. No. 115, January, 1942. Price 12s. 6d. each.

*Physical Signs in Clinical Surgery.* By Hamilton Bailey, F.R.C.S. Eighth Edition. Price 25s.

From General Medical Council :

*Fifth Addendum to the British Pharmacopœia,* 1932.

From Messrs. William Hodge & Co. Ltd. :

*The Life and Teaching of Sir William Macewen.* By A. K. Bowman. Price 21s.

From Henry Kimpton :

*Synopsis of the Preparation and Aftercare of Surgical Patients.* By Hugh C. Ilgenfritz, A.B., M.D., and Rawley M. Penick, Jr., Ph.B., M.D., F.A.C.S. Price 25s.

### Local Medical Notes

The thirty-first Long Fox Memorial Lecture was delivered, by kind permission of the Vice-Chancellor, in the Lecture Theatre of the H. H. Wills Physics Laboratory (Royal Fort), University of Bristol, on Tuesday, July 7th, at 8.30 p.m., by Professor C. M. Yonge, D.Sc., Professor of Zoology in the University of Bristol. Subject: "The Experimental Study of Development." Professor A. R. Short took the chair supported by three members of the Board.

EXAMINATION RESULTS.

**University of Bristol**—Students of the University have recently passed the following examinations:—

Ch.M. *Pass:* Coralie Rendle-Short.

M.B., Ch.B.—*Second Examination. Pass:* B. B. Chamberlain, J. R. Christie, W. P. Foster, A. S. Jones, F. D. Kay, C. J. Mulcahy, A. S. Nethercott, W. Oldham, D. V. Pleasant, R. M. Rocke, R. J. Sandry. *In Group II:* W. A. Heaton-Ward,*† H. M. Holt.† *In Group I only:* J. D. Medhurst.

M.B., Ch.B.—*Final Examination (Section I). Pass:* N. R. Baldwin, R. F. Brooks, R. M. Child, D. G. Davies (1, 2, 3), J. Deighton, M. D. Dixon, G. F. M. Hall (4, 5), D. Hughes, A. G. Mitchinson (4, 5), E. W. Moore, J. L. Pardoe, M. E. Seddon, E. S. Short, N. Slade, M. H. Thomas, G. H. Valentine, Jean Wallace, G. H. Wattley (1, 2, 3, 4, 5).

M.B., Ch.B.—*Final Examination. Pass:* J. L. Ashley, H. M. Hammond, K. M. Harland, P. Legat. *In Group I:* J. W. Brown,† J. A. Butt.† *In Group II:* S. J. V. Gilson.† *In Group I only:* H. A. M. Oakes. *In Group II only:* Jocelyn Brodie.

B.D.S.—*Second Examination. Pass:* H. L. Leech.

B.D.S.—*Third Examination. Pass:* C. R. W. Birkett,† J. B. K. Mann,*† I. A. Morgan.*

B.D.S.—*Final Examination. Pass:* (*In Section II*) (Dental Surgery), T. M. Nicholas† (*Second Class Honours*), F. C. Bryant,† P. B. Carey,† Sarah Jones.†

L.D.S.—*Third Examination. Pass:* H. B. Doubt,*† R. E. May,*† R. H. Shipway,*† R. C. Taylor,*† J. B. Westlake,*† F. E. R. Williams,*† H. Sheffield.*

L.D.S.—*Final Examination. Pass:* (*In Section II*) (Dental Surgery), J. M. Bryant,† V. T. Scard.† *In Section I only:* W. D. Arnold, P. M. Burke, G. C. Davis, G. Hellings, D. A. Holmes.

**University of Oxford.**—B.M., B.CH.—*Second Examination* (*a*) Special and Clinical Pathology, (*b*) Forensic Medicine and Public Health. *Pass :* Sheila R. Tangye.

**University of Cambridge.**—M.B.—*Final Examination. Part I* (Surgery, Midwifery and Gynæcology) *Examined and Approved :* Mrs. P. Eskell, D. S. Short.

**Society of Apothecaries, London.**—*Final Examination. Pass :* D. I. Fullerton (Medicine and Surgery).

**Royal Colleges of Physicians and Surgeons.**—L.R.C.P., M.R.C.S.—*First Examination. Pass :* W. F. Baptiste, (6, 7), R. W. E. Brain (7), F. J. Goddard (8), W. G. H. Hunt (8), P. R. Miller (6).

L.R.C.P., M.R.C.S.—*Final Examination. Pass :* B. C. E. Aldous† (9, 10), Margot H. Dickson (10, 11), F. Frank† (9), D. I. Fullerton, (9, 10), F. J. Goddard (12), A. J. R. Hudson (11), D. J. Jones (12), D. S. Short (11), G. Steiner (10), G. H. Valentine (9, 11, 12), E. L. Ward (11), G. H. Wattley (9, 11, 12), J. I. Williams (10).

DIPLOMA IN CHILD HEALTH.—*Pass :* J. L. Emery.

L.D.S., R.C.S.—*Final Examination. Pass. Part I :* S. Phillips, *Parts I and II :* F. C. Bryant.

**Diploma of the Royal College of Obstetricians and Gynæcologists.**—*Pass :* Dorothy M. Shotton.

* In Dental Mechanics and the Properties of Materials used in Dental Practice.

† Completing the Examination.

(1) With Distinction in Materia Medica.
(2) With Distinction in Pharmacy.
(3) With Distinction in Pharmacology and Toxicology.
(4) With Distinction in Pathology.
(5) With Distinction in Bacteriology.
(6) Anatomy. (7) Physiology. (8) Pharmacology.
(9) Medicine. (10) Surgery. (11) Midwifery. (12) Pathology.

### Bristol Medico-Chirurgical Society.

### President.

C. CORFIELD, M.D. Durh.

### President-Elect.

### Committee.

*Ex-Officio*

- J. A. NIXON, *C.M.G.*, B.A., M.D. Cantab., F.R.C.P. (Editor of *Journal*).

- (Vacant), (Hon. Librarian).

*Elected.*

L. A. MOORE, M.B., Ch.B. Brist. (Ex-President).
R. C. CLARKE, *O.B.E.*, M.B., Ch.B.Brist.
H. H. CARLETON, M.A., M.D., B.Ch.Oxon.
A. L. TAYLOR, M.D. Lond.
P. S. MARTIN, *M.C.*
T. MILLING, M.B., Ch.B. Belf.
W. V. WOOD, *M.C.*, B.A. Cantab.
H. L. SHEPHERD, M.B., Ch.M. Brist.
W. A. JACKMAN, F.R.C.S.

### Honorary Secretary.

R. V. COOKE, Ch.M.

### Honorary Treasurer.

A. E. ILES, *O.B.E.*, M.B., B.S. Lond., F.R.C.S.

### University Library Sub-Committee.

A. R. SHORT, B.Sc., M.D. Lond., F.R.C.S.
C. BRUCE PERRY, M.D. Brist., F.R.C.P.
H. J. DREW SMYTHE, *M.C.*, M.S., M.D. Lond., F.R.C.S., F.C.O.G.

Medical Practitioners wishing to join the Society are requested to communicate with the Hon. Secretary, Mr. R. V. COOKE, Failand Lodge, Guthrie Road, Clifton. The Annual Subscription is £1 1s., payable to Hon. Treasurer.

---

*Extracts from Journal By-laws.*

4.—The *Journal* shall be delivered free to Subscribers and also to Members of the Society whose subscriptions are not in arrear.

6.—The Annual Subscription to the *Journal* is 9 /–, payable to Hon. Treasurer.

---

Communications intended for insertion in the *Journal* should be sent to the Editor, Dr. J. A. NIXON, 7 Lansdown Place, Clifton, Bristol.

Books for Review and Exchange Journals should be sent to the Assistant Editor, *Bristol Medico-Chirurgical Journal*, Medical Library, The University, Bristol. Reviews should be returned with the books to Dr. T. M. LING, 50 Pembroke Road, Bristol 8.

Communications referring to the delivery of the *Journal* should be sent to the Hon. Treasurer, Mr. A. E. ILES, 87 Pembroke Road, Bristol 8.

Volumes of the *Journal* can be bound upon application to J. W. ARROWSMITH LTD., Quay Street, Bristol, price on request. The inclusive index for Volumes I—L may be obtained, price 3/– (postage extra).

### Bristol Medico-Chirurgical Society.

### PAST PRESIDENTS.

1874—75. FREDERICK BRITTAN, M.D., B.S. Dub.
1875—76. ROBERT WILLIAM COE.
1876—77. SAMUEL MARTYN, M.D. Ed.
1877—78. AUGUSTIN PRICHARD, M.D. Berlin.
1878—79. HENRY EDWARD FRIPP, M.D. Würzburg.
1879—80. WILLIAM MICHELL CLARKE.
1880—81. JOSEPH GRIFFITHS SWAYNE, M.D. Lond.
1881—82. EDWARD LONG FOX, M.D. Oxon.
1882—83. JAMES GEORGE DAVEY, M.D. St. And.
1883—84. WILLIAM JOHNSTONE FYFFE, M.D., B.A. Dub.
1884—85. GEORGE FORSTER BURDER, M.D. Aberd.
1885—86. WILLIAM HENRY SPENCER, M.D., M.A. Cantab.
1886—87. FRANCIS POOLE LANSDOWN.
1887—88. LEMUEL MATTHEWS GRIFFITHS.
1888—89. ROBERT SHINGLETON SMITH, M.D., B.Sc. Lond.
1889—90. NELSON CONGREVE DOBSON, Ch.M. Brist.
1890—91. SAMUEL HENRY SWAYNE.
1891—92. FRANCIS RICHARDSON CROSS, M.B. Lond., LL.D. Brist.
1892—93. EDWARD MARKHAM SKERRITT, M.D., B.S., B.A. Lond.
1893—94. JAMES GREIG SMITH, M.B., C.M., M.A. Aberd., F.R.S.E.
1894—95. ALFRED JAMES HARRISON, M.B. Lond.
1895—96. ARTHUR WILLIAM PRICHARD.
1896—97. ALFRED EDWARD AUST LAWRENCE, M.D., C.M. Aberd.
1897—98. JOHN EDWARD SHAW, M.B., C.M. Ed.
1898—99. ROBERT ROXBURGH, M.B. Ed.
1899—00. WILLIAM HENRY HARSANT.
1900—01. DAVID SAMUEL DAVIES, M.D. Lond.
1901—02. BARCLAY JOSIAH BARON, M.B., C.M. Ed.
1902—03. GEORGE MUNRO SMITH, M.D. Brist.
1903—04. JAMES PAUL BUSH, C.M.G., C.B.E., Ch.M. Brist
1904—05. REGINALD EAGER, M.D. Lond.
1905—06. JOHN DACRE.
1906—07. JAMES TAYLOR.
1907—08. HENRY WALDO, M.D., C.M. Aberd.
1908—09. JOHN MICHELL CLARKE, M.D., M.A. Cantab.
1909—10. JAMES SWAIN, C.B., C.B.E., M.D., M.S. Lond.
1910—11. BERTRAM MITFORD HERON ROGERS, M.D., B.Ch., B.A. Oxon.
1911—12. CHARLES ALEXANDER MORTON.
1912—13. WALTER CARLESS SWAYNE, M.D., B.S. Lond. and Brist.
1913—14. PATRICK WATSON-WILLIAMS, M.D. Lond., M.D. (Hon. Causa) Brist.
1914—15. WILLIAM HARRY CHRISTOPHER NEWNHAM, M.A., M.B. Cantab.
1915—16. GEORGE PARKER, M.A., M.D. Cantab., LL.D. Brist.
1916—17. FRANCIS HENRY EDGEWORTH, M.A., M.D. Cantab., D.Sc. Lond.
1917—18. FREDERICK LACE.
1918—19. ROBERT GUTHRIE POOLE LANSDOWN, M.D., B.S. Durh.
1919—20. I. WALKER HALL, M.D., Ch.B. Vict.
1920—21. LEOPOLD ERNEST VALENTINE EVERY-CLAYTON, M.D. Lond.
1921—22. CYRIL HUTCHINSON WALKER, B.A., M.B. Cantab.
1922—23. JOHN ALEXANDER NIXON, C.M.G., B.A., M.D. Cantab.
1923—24. JOHN LACY FIRTH, M.D., M.S. Lond.
1924—25. JOHN ODERY SYMES, M.D. Lond.
1925—26. THOMAS CARWARDINE, M.S. Lond.
1926—27. ARTHUR LAUNCELOT FLEMMING, M.B., Ch.B. Brist.
1927—28. WALTER KENNETH WILLS, M.A., M.B., B.Ch. Cantab.
1928—29. HENRY LAWRENCE ORMEROD, M.D., B.Ch. R.U.I.
1929—30. CHARLES FERRIER WALTERS.
1930—31. DAVID CHARLES RAYNER, Ch.M. Brist.
1931—32. JOHN ROGER CHARLES, M.A., M.D. Cantab.
1932—33. ERNEST WILLIAM HEY GROVES, M.D., M.S., D.Sc., B.Sc. Lond.
1933—34. HERBERT ELWIN HARRIS, B.A., M.B. Cantab.
1934—35. A. RENDLE SHORT, B.Sc., M.D. Lond.
1935—36. ARTHUR ERNEST ILES, O.B.E., M.B., B.S. Lond.
1936—37. RICHARD CHRISTOPHER CLARKE, O.B.E, M.B. Lond.
1937—38. CLIFFORD A. MOORE, M.B., M.S. Lond.
1938—40. LEONARD AUGUSTUS MOORE, M.B., Ch.B. Brist.
1940—41. CHARLES CORFIELD, M.D. Durh., Barr.-at-Law.
1941—42.

### LIST OF SUBSCRIBERS

TO THE

### Bristol Medico-Chirurgical Journal.

### HONORARY MEMBER OF THE SOCIETY.

Swain, James, *C.B.*, *C.B.E.*, M.D., M.S. Lond. .. .. .. Wyndham House, Easton-in-Gordano.

### SUBSCRIBERS.

*Those marked * are Members of the Society.*

| | |
|---|---|
| *Adams, A. W., M.S. Lond. .. .. .. .. | Elton House, Clifton, Bristol 8. |
| Adams, J. B. .. .. .. .. .. .. .. .. | Kent Lodge, Eastbourne. |
| *Adams, S.B., Ph.D., M.B., Ch.B. Brist. .. .. | 19 Charlotte Street, Bristol 1. |
| *Aldwinckle, Helen M., M.B., Ch.B. Brist. .. | 23 Rockland Road, Downend, Bristol. |
| *Alexander, A. J. P., M.D. Belf. .. .. .. | Winsley Sanatorium, near Bath. |
| *Alexander, D. A., M.B., Ch.B. Vict. .. .. | 112 Pembroke Road, Clifton, Bristol 8. |
| Alexander, G. L., B.A., M.B., B.Ch. Cantab. | West African Medical Service, Laura, via Accra, Gold Coast. |
| *Alford, A. F., M.B., Ch.B. Brist. .. .. .. | Board of Education, Durley Dene Hotel, Bournemouth. |
| Alford, H. J., M.D. Lond. .. .. .. .. | 4 Park Terrace, Taunton. |
| Armstrong, Jean, M.B., Ch.B. Brist. .. .. | Succoth, Duckmoor Road, Ashton Gate, Bristol 3. |
| Ashton, L. P., M.B., Ch.B. Brist. .. .. .. | Kapsowar, P.O. Eldoret, Kenya, East Africa. |
| *Aubrey, T., M.B. Lond. .. .. .. .. .. | Bitton, near Bristol. |
| *Bain, W. .. .. .. .. .. .. .. .. .. | 1 Greville Road, Southville, Bristol 3. |
| *Baker, Lily A., M.D., B.Ch., B.A.O., R.U.I. | 23 Pembroke Road, Clifton, Bristol 8. |
| *Barber, M. C., M.B., Ch.B. Brist. .. .. .. | Ingledene, High Street, Staple Hill, Bristol. |
| *Barbour, R. F., M.A., M.B., Ch.B. Cantab. | 16 Mortimer Road, Clifton, Bristol. |
| *Barker, G. H., M.D., B.Sc. Lond. .. .. .. | 124 Redland Road, Bristol 6. |
| *Bates, R. M. .. .. .. .. .. .. .. .. | Purdown House, Stapleton, Bristol 5. |
| *Baxter, Ursula, M.B., Ch.B. Brist. .. .. | 167 Wells Road, Knowle, Bristol 4. |
| *Baxter, V. T., M.B., Ch.B. .. .. .. .. | 167 Wells Road, Knowle, Bristol 4. |
| *Beatson, D. H., M.B., Ch.B. Brist. .. .. | 8 Richmond Park Road, Clifton, Bristol 8. |
| *Bell, R. J. Irving .. .. .. .. .. .. .. | 5a Oakfield Road, Clifton, Bristol 8. |
| *Bergin, F. G. .. .. .. .. .. .. .. .. | 31 Oakfield Road, Clifton, Bristol 8. |
| *Berry, R. J. A., Prof., M.D., C.M. Edin. .. | 320 Canford Lane, Bristol 9. |
| *Birrell, J. A., M.D., B.S. Lond. .. .. .. | 1 Victoria Square, Bristol 8. |
| Blackett, J. F., M.D., B.S. Lond. .. .. .. | Hamsterley, Bloomfield Park, Bath. |
| Bletchly, G. Playne, M.B. Lond. .. .. | c/o Lloyds Bank, Nailsworth, Glos. |
| *Bodman, A. P., M.B., Ch.B. .. .. .. .. | 11 Hurle Crescent, Clifton, Bristol 8. |
| *Bodman, F. H., M.D., Ch.B. Brist. .. .. .. | 2 Redland Court Road, Bristol 6. |
| Bolt, R. F. .. .. .. .. .. .. .. .. .. | 70 Great Russell Street, London, W.C.1. |
| *Boulton, B. J., M.B., Ch.B. Brist. .. .. | Ham Green Hospital, Pill, near Bristol. |
| *Bown, Naomi J., M.B., Ch.B. Brist. .. .. | The Corner, Nailsea, Somerset. |
| *Bradbeer, E. G., M.B., Ch.B. Brist. .. .. | 13 Percival Road, Bristol 8. |
| Brasher, C. W. J., M.D. .. .. .. .. | Murivance, Nether Stowey, Somerset. |
| *Brinckman, Winifred P., M.B., Ch.B. Brist. | 2 Clifton Park, Bristol 8. |
| *Britton, R. B., M.B., B.S. Lond. .. .. .. | 33a Whiteladies Road, Clifton, Bristol 8. |
| *Brocklehurst, R. J., M.A., D.M. Oxon. .. | The University, Bristol 8. |
| Brown, J. E., M.B., Ch.B. Brist. .. .. .. | Coulthurst Villa, Brives Road, Maraval, Trinidad, B.W.I. |
| Bryan, C. .. .. .. .. .. .. .. .. .. | 44 Nevil Road, Bishopston, Bristol 7. |
| *Burges, R. .. .. .. .. .. .. .. .. | Druid's Mead, Stoke Bishop, Bristol 9. |
| *Bush, G. B., M.B., Ch.B. Brist. .. .. .. | Litfield House, Clifton, Bristol 8. |

| | |
|---|---|
| *CAIRNS, F. J. | Devon House, Whitehall Road, Bristol 5. |
| CAMERON, MARGARET, M.B., Ch.B. | 1 Dean Lane, Bristol 3. |
| *CAPPER, W. M., M.B., B.S. Lond. | Litfield House, Clifton, Bristol. |
| *CAREY, R. S., *O.B.E.*, B.A. Cantab. | 502 Bath Road, Brislington, Bristol 4. |
| *CARLETON, H. H., M.A., M.D., B.Ch. Oxon. | 1 Lansdown Road, Clifton, Bristol 8. |
| *CASSON, E., M.D., Ch.B. Brist. | Mount Pleasant, Clevedon. |
| *CATES, H. J., M.D. Lond. | Northwoods House, Winterbourne, Bristol. |
| *CHAMBERS, E. R. | 9 Clifton Park, Clifton, Bristol 8. |
| *CHARLES, J. R., M.A., M.D. Cantab. | Litfield House, Clifton Down, Bristol 8. |
| *CHITTY, H., M.S. Lond. | Naish House, Clapton-in-Gordano. |
| *CHRISTOFFERSON, P. E., M.B., Ch.B. Brist. | 14 Oakfield Road, Clifton, Bristol 8. |
| *CLAREMONT, L. E., M.D.S. Brist. | 5 Rodney Place, Clifton, Bristol 8. |
| *CLARK, DENNIS O. | 168 Milton Road, Bristol Road, Weston-super-Mare. |
| *CLARKE, R. C., *O.B.E.*, M.B., Ch.B. Brist. | Harley Lodge, Clifton Park, Bristol 8. |
| *CLUTTERBUCK, E. R., M.B., Ch.B. Brist. | 63 Walliscote Road, Weston-super-Mare. |
| *COATES, H. | Notts County Mental Hospital, Radcliffe-on-Trent. |
| *COCHRANE, J. H. | 11 Bryant's Hill, St. George, Bristol 5. |
| *COLLINGWOOD, F. C., M.B., Ch.B. Brist. | 7 Hurle Crescent, Clifton, Bristol 8. |
| *COOKE, ELIZABETH M., M.D. Lond. | Failand Lodge, Guthrie Road, Clifton. |
| *COOKE, R. V., Ch.M. Brist. | Litfield House, Bristol 8. |
| *COOKSON, R. G. | Clifton College, Erdiston, Bude. |
| COOMBS, N. C. | Romaldkirk, Barnard Castle, Co. Durham. |
| *COOPER, W. R. | 13 Westfield Park, Redland, Bristol 6. |
| *CORFIELD, C., M.D. Durh. | 5 Kensington Place, Clifton, Bristol 8. |
| *CORNER, BERYL, M.D., B.S. Lond. | 3 Elton House, Rodney Place, Bristol 8. |
| *COSSHAM, W. L., M.B., Ch.B. Brist. | 3 Claremont Road, Bishopston, Bristol 7. |
| *COURTNEY, R. M., M.B., Ch.B. Glasg. | Manor House, Southmead Road, Bristol |
| *COXON, BEATRICE | 16 Richmond Hill, Clifton, Bristol 8. |
| *CRAIG, ANNE A., M.D., B.S. Lond. | 35 Queen's Court, Clifton, Bristol 8. |
| *CROOK, B. A., M.B., Ch.B. Brist. | The Laurels, Timsbury, near Bath. |
| CROOT, H. J., M.B., B.S. Lond. | Royal Infirmary, Bristol 2. |
| *CROSSMAN, F. W., M.B. Durh. | White's Hill, Hambrook, near Bristol. |
| *DATTA, S. S., M.D. Brist. | Snowdon House, Fishponds, Bristol. |
| *DAVIES, E. D. D. | Standish House, Stonehouse, Glos. |
| *DAVIES, J. N. P., M.B., Ch.B. | Physiology Department, The University, Bristol. |
| *DELANEY, N., M.B., Ch.B. Manch. | The Meade, Bishopsworth, Somerset. |
| *DICKSON, W. A., M.D. St. And. | Standish House, Stonehouse, Glos. |
| *DISNEY, M., M.D.Lond. | 2 Clifton Park, Bristol 8. |
| *DIX, C. | 14 Mortimer Road, Clifton, Bristol 8. |
| *DIXON, T. B. | 11 Pembroke Road, Clifton, Bristol 8. |
| *DODD, GRANTHAM, M.B., B.S. | 42 Woodstock Road, Bristol 6. |
| *DUERDEN, J. R., M.B., Ch.B. Brist. | 4 Cecil Road, Bristol 8. |
| *DUTTON, HILDA R. | 405 Stapleton Road, Bristol 5. |
| *DYER, W. P. | West View, Westbury-on-Trym, Bristol. |
| *EGLINTON, J. H. C. | Street, Somerset. |
| ELLIOTT, F. P., M.B., C.M. Aberd. | 113 Grove Road, Walthamstow, London, E. 17. |
| *ELLIOTT, W. H. A., M.B., B.S. Lond. | 29 Old Sneed Avenue, Stoke Bishop, Bristol 9. |
| *ELLIS, W. T. | Tregenna House, Shirehampton. |
| *EMERY, J. L. | Childrens' Hospital, Bristol 2. |
| *EVANS, G. M., M.B., Ch.B. Brist. | 117 Ashley Road, Bristol. |
| *EYRE-BROOK, A. L., M.B., M.S. Lond. | The Cottage, Portishead. |
| *FALLON, Col. J. | 35 Henleaze Gardens, Henleaze, Bristol. |
| *FAWN, G. F. | 6 Oakfield Road, Clifton, Bristol 8. |
| *FELLS, G. R., M.B., Ch.B. Brist. | 19 Southmead Road, Bristol. |
| *FELLS, R. R., M.B., B.S. Lond. | 17 Mortimer Road, Clifton, Bristol 8. |
| *FENELEY, G. L., M.B., Ch.B. Brist. | Englewood, Falcondale Road, Westbury-on-Trym. |

| | |
|---|---|
| *FINZEL, H. F. M., M.D. Brist. .. .. .. .. .. | Nare House, Birchwood Road, St. Anne's Park, Bristol 4. |
| *FIRTH, J. L., M.D., M.S. Lond. .. .. .. .. | 8 Victoria Square, Clifton, Bristol 8. |
| FISCHER, I. .. .. .. .. .. .. .. .. .. .. .. | 32 Kewstoke Road, Stoke Bishop, Bristol 9. |
| *FISHER, A. C., M.D. Brist. .. .. .. .. .. | Roan Antelope Mine, N. Rhodesia. |
| *FITZGIBBON, G. M., M.D. Lond. .. .. .. .. | 75 Pembroke Road, Clifton, Bristol 8. |
| *FITZGIBBON, L. PRISCILLA, M.B., B.S. Lond. .. | 75 Pembroke Road, Clifton, Bristol 8. |
| *FORRESTER-BROWN, MAUD, M.D., M.S. Lond. | 22 Combe Park, Bath. |
| *FOSS, G. L., M.B., B.Ch. Cantab. .. .. .. .. | Cloud's Hill House, St. George, Bristol 5. |
| *FOX, F. E., B.A. Cantab. .. .. .. .. .. .. | Brislington House, Brislington, Bristol 4. |
| *FRASER, A. D., M.A., M.B., Ch.B. Glasg. .. | 4 Alexandra Road, Clifton, Bristol 8. |
| *FRASER, D., M.B., Ch.B. Edin. .. .. .. .. | Westgate Dursley, Glos. |
| *FULLER, H. L. .. .. .. .. .. .. .. .. .. .. | 19 The Circus, Bath. |
| *FURNIVAL, Col. C. H. .. .. .. .. .. .. .. | " Yatton," Wellington Hill Horfield, Bristol 7. |
| *GARDEN, R. R., M.A., M.B., B.Ch. Aberd. .. | 9 Harley Place, Clifton, Bristol 8. |
| GARLAND, E. B., M.B., C.M. Edin. .. .. .. .. | 114a Hampton Road, Redland, Bristol 6. |
| *GERRISH, NORMAN, M.B., Ch.B. Brist. .. .. | 2 Station Road, Keynsham, Som. |
| *GIBBS, J. R. .. .. .. .. .. .. .. .. .. .. .. | 48 Codrington Road, Bishopston, Bristol 7. |
| *GORHAM, A. P., M.B., Ch.B. Brist. .. .. .. | 53 Westbury Road, Westbury-on-Trym. |
| *GORNALL, W. A., M.D. Brist. .. .. .. .. .. | Ulvik, Nailsea, near Bristol. |
| *GREEN, S. B., M.B. Lond. .. .. .. .. .. .. | The Black Wicket, Westbury-on-Trym. |
| *GREENLAW, MADGE E., M.B., Ch.B. Brist. .. | 206 Redland Road, Bristol 6. |
| GRIFFITHS, CORNELIUS A. .. .. .. .. .. .. | 35 Newport Road, Cardiff. |
| *GROVES, E. W. HEY, M.D., M.S., D.Sc. Lond. .. | 25 Victoria Square, Clifton, Bristol 8. |
| *HALL, E. GEORGE, M.B. Lond. .. .. .. .. .. | 42 Apsley Road, Clifton, Bristol 8. |
| *HAM, C. I., M.B., Ch.B.Brist. .. .. .. .. .. | c/o Dr. Craig, Sedbergh Road, Kendal. |
| *HARRIS, H. E., M.A., M.B., B.Ch. Cantab. .. | 13 Lansdown Place, Clifton, Bristol 8. |
| *HARTLEY, GRACE, M.B., B.S. Lond. .. .. | 49 Queen's Court, Clifton, Bristol 8. |
| *HEALES, J. N., M.B., Ch.B. Brist. .. .. .. | Holmcroft, Street, Somerset. |
| *HECTOR, F., M.D. Lond. .. .. .. .. .. .. .. | 1 Victoria Square, Clifton, Bristol 8. |
| *HEMPHILL, R., M.D. Dub. .. .. .. .. .. | 90 Cleeve Hill, Downend, near Bristol. |
| *HENDERSON, JAMES, M.B., Ch.B. Aberd. .. | The City Sanatorium, Yardley Road, Birmingham. |
| *HERAPATH, C. E. K., *M.C.*, M.D.Lond. .. | Ormlie, Clifton Down, Bristol 8. |
| *HEWER, Professor T. F., M.D. Brist. .. .. | Canynge Hall, Bristol 8. |
| *HILL, WINIFRED, M.B., Ch.B. Brist. . .. | c/o Lloyd's Bank, Ilford. |
| *HOFFMAN, H. LOVELL, B.A., M.B., B.Ch. Cantab. | R.N. Hospital, Invergordon. |
| *HOLLAND, W. A. L. .. .. .. .. .. .. .. .. | 10 Brunswick Square, Bristol. |
| *HOOKER, J. A., Colonel, M.B., Ch.B.Brist. .. | 16 St. Edyth's Road, Sea Mills 9. |
| *HOSKEN, J. G. F... .. .. .. .. .. .. .. .. | Uley, Glos. |
| HOSPITAL, BRISTOL GENERAL (*Secretary*, T. W. | GREGG). |
| *HOUGHTON, LYDIA M., M.B., B.S. Lond. .. | 74 Church Road, Redfield, Bristol 5. |
| *HOWELL, H. .. .. .. .. .. .. .. .. .. .. .. | Kincraig, Beach Road, Weston-super-Mare. |
| *HOWELL, T. E. .. .. .. .. .. .. .. .. .. .. | 6 Richmond Hill, Bristol 8. |
| *HUGHES, F. W. TERRELL, M.B., Ch.B. Brist. | Chew Magna, Somerset. |
| *HUGHES, MARGUERITE G., M.B., Ch.B. Brist. | 12 Upper Belgrave Road, Clifton, Bristol 8. |
| *HUNTER, F. S. .. .. .. .. .. .. .. .. .. | c/o Messrs. J. Wright & Sons Ltd., Publishers, 28 Orchard Street, Bristol 1. |
| *HYATT, ANNIE W., M.B., B.S. Lond. .. .. | Merrymead, Shepton Mallet, Som. |
| *ILES, ANNA, M.B., Ch.B. Brist. .. .. .. .. | 87 Pembroke Road, Clifton, Bristol 8. |
| *ILES, A. E., *O.B.E.*, M.B., B.S. Lond. .. .. | 87 Pembroke Road, Clifton, Bristol 8. |
| INFIRMARY, BRISTOL ROYAL (*Secretary*, E. C. | SMITH). |
| INGLE, C. DURBAN .. .. .. .. .. .. .. .. | Somerton, Somerset. |
| *ISSERLIN, B. .. .. .. .. .. .. .. .. .. .. .. | 2 College Road, Bristol 8. |
| *JACKMAN, W. A., *T.D.*, M.B., Ch.B. Brist. .. | 15 Mortimer Road, Clifton, Bristol 8. |
| *JAMES, APRIL D., M.B., Ch.B. Brist. .. .. | (Address unknown.) |
| *JAMES, J. A., M.D. Lond. .. .. .. .. .. .. | Litfield House, Clifton Down. Bristol 8. |
| *JAMES, J. A., Senr. .. .. .. .. .. .. .. .. | Dusk, Elmlea Avenue, Stoke Bishop, Bristol, 9. |

| | |
|---|---|
| *Jenkins, F. G., M.B., Ch.B. Brist. | 51 Redcliff Hill, Bristol 1. |
| *Jenkins, R. D. | The Copper Beeches, Almondsbury, near Bristol. |
| *Jenkins, P. K., M.B., Ch.B. | R.A.M.C. |
| *Johnson, R. G., M.B. Lond. | 119 Redland Road, Bristol 6. |
| *Joll, C. A., M.D., M.S. Lond. | 64 Harley Street, London, W.1. |
| *Jones, Megan E. | Royal Infirmary, Bristol 2. |
| *Jordan, L. R., M.B., Ch.B. Brist. | National Temperance Hospital, Hampstead Road, N.W.1. |
| *Joscelyne, P. C., M.B., Ch.B. Brist. | 17 Falcondale Road, Westbury-on-Trym. |
| *Kemm, N. F., M.B., B.S. Lond. | 200 Redland Road, Durdham Park, Bristol, 6. |
| *Kersley, G. D., M.D. | 6 The Circus, Bath. |
| King, E. F. | 2 Upper Wimpole Street, London, W.1. |
| *Kingston, S. H., M.B., Ch.B. Brist. | 3 Whiteladies Road, Clifton, Bristol 8. |
| *Kyle, H. G., M.A., M.D., B.Ch. Oxon. | 31 Westbury Road, Bristol. |
| *Lace, F | 5 Gay Street, Bath. |
| Lalonde, T. P., M.B., Ch.B. Brist. | Wykeham House, Romsey, Hants. |
| *Lees, E. Leonard, M.D., C.M. Edin. | 28 Downs Park West, Bristol 6. |
| *Legat, R. Eddowes, M.B., C.M. Edin. | Hampden, St. Briavels, Glos. |
| *Lewis, F. J., M.B., Ch.B. Brist. | Canynge Hall, Bristol 8. |
| *Ling, T. M., M.A., B.M., B.Ch. | 50 Pembroke Road, Bristol 8. |
| *Linton, Marion S., M.B. Lond. | 1 Brecon Road, Henleaze, Bristol. |
| *Lister, A. E. J., M.B., B.S. Lond. | 86 Pembroke Road, Clifton, Bristol 8. |
| *Logan, H. B. | Elmwood, Aberdeen Road, Cotham, Bristol 6. |
| *Loxton, S. D., M.B., Ch.B. | 17 Oakfield Grove, Clifton, Bristol 8. |
| *Lucas, J. E. | c/o 132 York Road, Bristol 3. |
| *Lucas, J. J. S., M.D. Lond. | 186 Wells Road, Knowle, Bristol 4. |
| *Lucas, J. V. | 186 Wells Road, Knowle, Bristol 4. |
| *Lucas R. P., M.B., Ch.B. Brist. | 15 Edgecumbe Road, Redland Bristol 6. |
| *McKenzie, W. G., *M.C.* | 11 Bath Road, Reading. |
| *MacLachlan, A. M., M.B., Ch.B. Glasg. | 65 Redcliff Hill, Bristol 1. |
| *Marwood, S. F., M.D., B.S. | Troy House, Kingswood, Bristol. |
| *McLannahan, J. G. | The Mount, Stonehouse. |
| *MacLaren, Catherine J., M.B., Ch.B. Edin. | Hereford House, Clifton Down. |
| Maclean, Sir Ewen J., M.D., C.M. Edin. | 12 Park Place, Cardiff |
| *Macleod, G., M.A. Glas., M.D., M.B. | Wargrave, Clevedon, Somerset. |
| *Marshall, A. H., M.B., Ch.B. Brist. | Odelos, Canford Lane, Bristol. |
| Marshall, A. L., M.B., B.A. Cantab. | Laxfield, Woodbridge, Suffolk. |
| *Martin, P. S., *M.C.* | Victoria Quadrant, Weston-super-Mare. |
| *Matheson, N. R., M.B., Ch.B. | 37 Abbey Road, Westbury-on-Trym. |
| Miall, C. L'O | Elm Hayes, Paulton, near Bristol. |
| *Milling, T., M.B., Ch.B. Belf. | 1 Vicarage Road, Southville, Bristol 3. |
| *Moore, C. A., M.B., M.S. Lond. | South Lodge, Kingsweston Road, Henbury Bristol. |
| *Moore, L. A., M.B., Ch.B. Brist. | Hillsborough, Cotham Park, Bristol 6. |
| *Moore, R. H., M.B., Ch.B. Brist. | White Cross Court, Wells Road, Bristol 4. |
| *Morgans, C. C., M.B., Ch.B. Cantab. | 65 Lower Redland Road, Bristol 6. |
| *Morris, L. N., M.B., Ch.B. Brist. | 13 Belgrave Road, Clifton, Bristol 8. |
| *Mundy, G. S., M.B., B.Ch. Brist. | Homewood, Coombe Dingle, Bristol. |
| *Myles, Q. St. L., M.A., B.M., B.Ch. Oxon. | 7 Apsley Road, Clifton, Bristol 8. |
| *Nicholson-Lailey, J. R. | Elmfield House, South Road, Taunton. |
| *Nixon, Doreen, M.B., B.S. Lond. | 7 Lansdown Place, Clifton, Bristol 8. |
| *Nixon, J. A., *C.M.G.*, M.D. Cantab. | 7 Lansdown Place, Clifton, Bristol 8. |
| *Noble, J. I., M.B., Ch.B. Liverp. | 102 Pembroke Road, Clifton, Bristol 8. |
| *Norman, R. M., M.D. Brist. | Highwood, Stapleton Park, Bristol. |
| *Nott, Winifred, M.B., Ch.B. Brist. | Fassiefern, 8 Rylestone Grove, Stoke Bishop, Bristol 9. |
| *O'Donohoe, Monica A., M.B., Ch.B. | 1 Beaconsfield Road, Bristol 8. |

| | |
|---|---|
| *ORMEROD, G. L. M.A., M.B., B.Ch. Cantab .. | 2 Henleaze Road, Westbury-on-Trym, Bristol. |
| *ORR-EWING, H. J., *M.C.*, M.D. Lond. .. .. | 1 Beaufort Buildings, Clifton, Bristol 8. |
| *PALIN, A. G., B.M., B.Ch. Oxon. .. .. .. | Litfield House, Clifton Down, Bristol 8. |
| *PARKES, J. A., M.B., Ch.B. Liverp. .. .. | 8 South Road, Redland, Bristol 6. |
| *PARKES, P. W. J. .. .. .. .. .. .. .. | 164 Filton Avenue, Bristol 7. |
| PARKINSON, Lt.-Col. G. S., *D.S.O.*, R.A.M.C. | London School of Hygiene and Tropical Medicine, Keppel Street, London, W.C. 1. |
| *PARRY, R. H., M.B., B.S.Lond. .. .. .. | Public Health Offices, Bristol 1. |
| PARSONS, Sir J. H., M.B., B.S., B.Sc. Lond... | 54 Queen Anne Street, London, W. |
| *PAUL, R. G. .. .. .. .. .. .. .. .. .. | 23 Pembroke Road, Bristol 8. |
| *PAULI, C. H... .. .. .. .. .. .. .. .. | Pagan's Hill Farm, Chew Stoke. |
| *PERRY, B., M.D., Ch.B.Brist. .. .. .. .. | Canynge Hall, Clifton, Bristol 8. |
| PHELPS, P. C., M.B., Ch.B. .. .. .. .. | 23 St. Alban's Road, Bristol 6. |
| *PHILLIPS, P., M.B., Ch.B. Brist. .. .. .. | Southmead Hospital, Bristol. |
| *PIRRIE, A. L., M.B., Ch.B. Glasg. .. .. | 528 Kensington Hill, Bristol. |
| *POCOCK, J. A., M.A., M.B., B.Ch. Cantab. .. | General Hospital, Bristol. |
| PORTER, C. P. .. .. .. .. .. .. .. .. | 28 Mill Street, Kidderminster. |
| *POTTER, MABEL F., M.B., Ch.B. Brist. .. .. | 79 Pembroke Road, Clifton, Bristol 8. |
| *POWELL, H. F., M.D. Brux. .. .. .. .. | Ellenborough Park, Weston-super-Mare. |
| *PRICE, C. H. G., M.B., Ch.B.Brist. .. .. | The Laurels, Chew Stoke. |
| *PRICE, N. L., M.B., Ch.B. Brist. .. .. .. | Litfield House, Clifton Down, Bristol 8. |
| *PRIDIE, K. H., M.B., B.S. Lond. .. .. .. | 58 St. John's Road, Clifton, Bristol 8. |
| *PROWSE, D. C., M.B., Ch.B. Brist. .. .. .. | Wigmore House, Thornbury, near Bristol. |
| *PYKE, H. D., M.B., Ch.B. Brist. .. .. .. | 88 Redland Road, Bristol 6. |
| *RANKIN, P. C., M.B., Ch.B. Glasg. .. .. .. | Lawrence Hill House, Bristol. |
| *RAYNER, D. C., Ch.M. Brist. .. .. .. .. | 9 Lansdown Place, Clifton, Bristol 8. |
| *ROBERTS, J. A. F., M.A. Cantab., M.B., Ch.B., D.Sc. Edin. | Stoke Park Colony, Bristol. |
| *ROBERTSON, D., M.B., Ch.B. Edin. .. .. .. | 2 Knowle Road, Knowle, Bristol 4. |
| *ROBERTSON, L. G., M.B., Ch.B. .. .. .. | 162 Redland Road, Bristol 6. |
| *ROGERS, H., M.D. Brist. .. .. .. .. .. | 91 Old Park Ridings, Grange Park, N.21. |
| *ROLFE, R., B.A., M.B., B.C. Cantab. .. .. | Park House, St. Andrew's Road, Avonmouth. |
| *ROWLANDS, R. D. .. .. .. .. .. .. | Connaught Military Hospital, Knaphill, Surrey. |
| *ROWLEY, A. T. .. .. .. .. .. .. .. .. | 13 Walliscote Road, Weston-super-Mare. |
| *RUDOLF, MAJOR G. DE M. .. .. .. .. | R.A.M.C. |
| *RYAN, P. B., M.B., Ch.B... .. .. .. .. | 43 Nevil Road, Bishopston, Bristol. |
| *SAMPSON, O. E. L., M.B., Ch.B. Brist. .. .. | 77 Stackpool Road, Bristol 3. |
| *SCARFF, G., M.B., Ch.B. Edin... .. .. .. | 83 Pembroke Road, Clifton, Bristol 8. |
| *SHEPHERD, H. L., M.B., Ch.M. Brist. .. .. | 79 Pembroke Road, Clifton, Bristol 8. |
| *SHORT, A. R., B.Sc., M.D., B.S.Lond... .. .. | 69 Pembroke Road, Clifton, Bristol 8. |
| *SMITH, T. W., M.D. Lond... .. .. .. .. | 6 Oakley, Claverton Down, Bath. |
| *SMYTHE, H. J. D., *M.C.*, M.D., M.S. Lond. .. | 15 Eaton Crescent, Clifton, Bristol 8. |
| *SNAWDON, J. W. E., M.B., Ch.B., B.D.S. .. .. | Litfield House, Clifton, Bristol 8. |
| *SOLTAU, H. K. V., M.D. Lond. .. .. .. | 19 The Avenue, Clifton, Bristol 8. |
| *SOMERS, MARY A. E. .. .. .. .. .. .. | 2 Ashcombe Road, Weston-super-Mare. |
| *SPOOR, A., M.B., B.Ch. Cantab. .. .. .. | The Hydro, Bristol 1. |
| *STEVEN, G. D., M.B., Ch.B. Edin. .. .. .. | 6 Upper Church Street, Bath. |
| *STEPHEN, R. J. .. .. .. .. .. .. .. | 92 Redland Road, Bristol 6. |
| STONE, DORIS M., M.D., B.S. Lond. .. .. | Beechwood, Church Street, Epsom. |
| *STROVER, H. W. M., *O.B.E.*, M.B., Ch.B. Aberd. | 90 Redland Road, Bristol 6. |
| *STRUTHERS, A. J., M.B., Ch.B. Glasg. .. .. | 100 Church Road, Redfield, Bristol 5. |
| *STRUTHERS, GEO., M.B., Ch.B. Glasgow .. .. | 100 Church Road, Bristol 5. |
| *SUTTON, G. E. F., M.D. Lond... .. .. .. | 1 Pembroke Road, Clifton, Bristol 8. |
| *SYMES, J. O., M.D. Lond... .. .. .. .. | 6 Pembroke Vale, Clifton, Bristol 8. |
| *TALLACK, R. J. K., M.B., Ch.B. .. .. .. | Park Hurst, Nore End, Portishead. |
| *TASKER, D. G. C., M.B., M.S. Lond. .. .. | 9 College Fields, Clifton, Bristol 8. |
| *TAYLOR, A. L., M.D. Lond. .. .. .. .. | The General Hospital, Bristol 1. |

| | |
|---|---|
| TAYLOR, A. L. .. .. .. .. .. .. .. .. | The Royal Infirmary, Bristol 2. |
| THIN, J. .. .. .. .. .. .. .. .. .. .. .. | 54 and 55 South Bridge, Edinburgh. |
| *TODD, A. T., *O.B.E.*, M.D. Edin. .. .. .. | Royal Park House, Clifton, Bristol 8. |
| TRINICK, RICHARD H. .. .. .. .. .. .. .. | 42 Cherry Valley Gardens, Belfast. |
| TRIPP, DOROTHY M. H. .. .. .. .. .. .. | Mission Hospital, Karim Nagar, Nizam's Dominions, South India. |
| *TRIST, J. R. R., *M.C.* .. .. .. .. .. .. | 120 Henleaze Road, Henleaze. |
| *TRYON, VICTORIA S., M.B., Ch.B. Brist. .. | 3 Harley Place, Clifton, Bristol 8. |
| *WAGSTAFFE, E. M., M.B., Ch.B. .. .. .. .. | Winford Orthopædic Hospital. |
| *WANSBROUGH, T. B., M.B., Ch.B. Brist. .. | 23 Pembroke Road, Clifton, Bristol 8. |
| *WALKER, CYRIL H., B.A., M.B. Cantab. .. | Harewood, Clapton-in-Gordano, Som. |
| *WALTERS, C. F. .. .. .. .. .. .. .. .. .. | Downover, Walton Down, near Clevedon. |
| WAKE, S. C., M.B., Ch.B. Brist. .. .. .. | "Stanhope," Bideford, N. Devon. |
| *WARD, H. W. .. .. .. .. .. .. .. .. .. | Chipping Manor, Wotton-under-Edge. |
| *WARREN, R., M.A., M.D. Oxon. .. .. .. | Heathgates, Weston-super-Mare. |
| *WATSON-WILLIAMS, E., *M.C.*, B.A., M.B. Cantab., Ch.M. Brist. | 65 Pembroke Road, Clifton, Bristol 8. |
| WATTS, J. B. V. .. .. .. .. .. .. .. .. .. | Frantzina's Rust, Barberton, Transvaal, S. Africa. |
| WEDDELL, C. W. .. .. .. .. .. .. .. .. .. | Boydville, 46 Miller Street, West Melbourne, Australia. |
| *WIGODER, SYLVIA, M.A., M.D., Ph.D. .. .. | General Hospital, Bristol 1. |
| *WILBOND, R. G. .. .. .. .. .. .. .. .. .. | 76 Chesterfield Road, St. Andrew's Pk., Bristol. |
| *WILLIAMS, I., M.B., Ch.B. Brist. .. .. .. | "Ardrem," Hill View, Henleaze, Bristol. |
| *WILLIAMS, S. R., M.B. Lond. .. .. .. .. | Buckfastleigh, Devon. |
| *WILLWAY, F. W., M.D., M.S. Lond. .. .. | Bristol Royal Infirmary. |
| *WOOD, D. .. .. .. .. .. .. .. .. .. .. .. | 105 Pembroke Road, Clifton, Bristol 8. |
| *WOOD, URSULA, M.B., Ch.B. .. .. .. .. | Henley Lodge, Yatton, Som. |
| *WOOD, W. V., *M.C.*, B.A. Cantab. .. .. .. | Henley Lodge, Yatton, Som. |
| *WOODMAN, DOROTHY, M.D. Lond. .. .. .. | Clifton Hill House. |
| *WOOLLEY, W., M.B., Ch.B. Brist. .. .. .. | 239 Cranbrook Road, Redland. |
| *WRIGHT, A. J. M., M.B., B.S. Lond. .. .. | Harley Cottage, Clifton Park, Clifton, Bristol 8. |
| *WRIGHT, WINIFRED M., M.B., B.S. Lond. .. | Merrymead, Shepton Mallet, Som. |
| *ZEALAND, L., M.D. Man. .. .. .. .. .. .. | Brecon Lodge, Westbury-on-Trym, Bristol. |

### *HONORARY VISITING MEMBERS.*

BRIDGES, MAJOR ARTHUR BRODIE HAMILTON, R.A.M.C., *O.B.E.*

WARREN, SURGEON REAR-ADMIRAL LEONARD, *O.B.E.*, M.B., B.S. Lond.

WHITBY, COLONEL LIONEL ERNEST HOWARD, *A.M.S.*, *C.V.O.*, *M.C.*, M.D. Cantab.

WORTHINGTON, COLONEL FRANK, *A.M.S.*, *D.S.O.*, *O.B.E.*, M.B., B.Ch. Cantab.

And their staffs.

